# DKK1 Ameliorates Myofibroblast Differentiation in Urethral Fibrosis in Vivo and in Vitro by Regulating the Canonical Wnt Pathway

**DOI:** 10.7150/ijms.79827

**Published:** 2023-09-25

**Authors:** Shanlong Huang, Delai Fu, Ziyan Wan, Zhixin Huang, Min Li, Hecheng Li, Tie Chong

**Affiliations:** 1 Department of Urology, The Second Affiliated Hospital of Xi'an Jiaotong University, 157 Xiwu Road, Xi'an, China.; 2 Department of Pediatrics, The Second Affiliated Hospital of Xi'an Jiaotong University, 157 Xiwu Road, Xi'an, China.

**Keywords:** urethral stricture, urethral fibrosis, Wnt pathway, myofibroblast differentiation, DKK1

## Abstract

**Background:** Urethral stricture is a common disorder of the lower urinary tract in men. A complex network of pathways and interactions are involved in the pathogenesis of urethral fibrosis. However, the mechanisms underlying urethral fibrosis remain poorly understood.

**Objectives:** To investigate the critical role of the canonical Wnt pathway in development of urethral fibrosis and explore DKK1, the endogenous inhibitor of Wnt pathway, as a potential target to prevent urethral fibrosis *in vitro* and *in vivo*.

**Methods:** Urethral fibrosis tissue derived from patients and rat models were harvested to assess the activation of the canonical Wnt pathway by using western blot, qRT-PCR and immunohistochemistryWe performed histological staining, western blot, qRT-PCR and immunohistochemistry to examine the effects of DKK1 treatment on *in vivo* rat urethral fibrosis models. *In vitro*, human urethral fibroblasts (HUFs) were cultured to examine the effects of DKK1 in TGFβ1-induced HUFs by CCK-8 assay, hydroxyproline assay, flow cytometry, cell migration assay, western blot, qRT-PCR and immunofluorescence.

**Results:** The key components of Wnt signaling were upregulated in urethral fibrosis tissue derived from patients and rat models while DKK 1 was downregulated. DKK1 ameliorated TGFβ1-induced urethral fibrosis in rats. TGFβ1 induced myofibroblast differentiation by upregulating collagen I, collagen III, α-SMA, β-catenin and p-GSK-3β, while DKK1 was decreased. DKK1 significantly inhibited cell proliferation, collagen content, cell migration and promoted cell apoptosis in TGFβ1-induced HUFs. DKK1 significantly suppressed myofibroblast differentiation of TGFβ1-induced HUFs by downregulating collagen I, collagen III, α-SMA, β-catenin and p-GSK-3β with a mechanism independent of Smad2/3.

**Conclusions:** Our study demonstrated that canonical Wnt pathway may be an essential mechanism underlying the pathogenesis of urethral fibrosis and explored the potential role of DKK1 participation in the development of urethral fibrosis.

## Introduction

Urethral stricture is a narrowing of the urethral lumen caused by excessive collagen deposition of spongio-fibrosis^1^. With high prevalence and recurrence, it is a great clinical challenge for both patients and urologists^2^, ^3^. Complications of urethral stricture have a significant impact on patients' quality of life and health-care costs^4^. Currently, surgical strategies are the main considerable treatment options for patients such as urethrotomy or urethroplasty. However, most of these postoperative patients have been plagued by high recurrence. The pathophysiological mechanisms underlying urethral stricture remain poorly understood despite extensive study. Urethral fibrosis has been shown to be the result of an abnormal accumulation and activation of myofibroblasts leading to overexpression of collagen and excessive deposition of extracellular matrix 5, 6. There exists an urgent medical need for exploring new mechanisms and novel potential targets for preventing urethral fibrosis.

Transforming growth factor β1 (TGFβ1) plays the primary factor in the pathogenesis of fibrosis. Recent studies have indicated the vital role of TGFβ1 in the pathophysiology of urethral stricture formation 7, 8. A number of signaling pathway regulate TGFβ signaling, resulting in a complex of interactions known as pathway crosstalk, including MAPK, Wnt, mTOR, Notch and other signaling^9^. Our previous studies have demonstrated that mTOR and Notch signaling contribute to TGFβ1-induced urethral fibrosis^10^, ^11^. In addition, canonical Wnt signaling is an evolutionarily conserved signaling pathway that has been suggested to be crucial in regulating developmental processes during embryogenesis and tissue homeostasis^12^. Particularly, accumulating evidence indicates that aberrant activation of the canonical Wnt pathway might play an important role in fibrosis^13^. Pathologically activated canonical Wnt pathway has been implicated in organ fibrosis diseases, including lung, kidney and liver fibrosis^14^-^16^. It has been reported that TGFβ can stimulate canonical Wnt signaling and activation of the canonical Wnt signaling could contribute to the profibrotic effects of TGFβ^17^. However, it remains unknown whether canonical Wnt signaling is activated in or contributes to urethral fibrosis. Therefore, unraveling this may have wide-ranging significance to exploring new targets for preventing urethral fibrosis.

β-catenin, the central signaling component of the canonical Wnt pathway, accumulates in the cytoplasm and translocate into the nucleus to mediate transcription of target genes when the canonical Wnt signaling is activated^18^. The canonical Wnt signaling is also controlled by endogenous and negative regulators, including Dickkopf proteins. The best studied member is Dickkopf1 (DKK1), which inhibits the canonical Wnt signaling by binding to LRP5/6 and blocking Wnt interactions^19^. DKK1 is involved in many physiological and pathological processes, including osteoporosis, Alzheimer's disease, diabetes, various cancers and fibrosis diseases^20^. Along this line, DKK1 seems to be a potential target to restrain activation of the canonical Wnt signaling to prevent urethral fibrosis.

In this study, we investigated the potential role of the canonical Wnt signaling in the pathophysiological development of urethral fibrosis and assessed whether inhibition of the canonical Wnt signaling with DKK1 could prevent urethral fibrosis *in vitro* and *in vivo*. The main findings of our study were the attenuation effects of DKK1 in urethral fibrosis, which helped to have a better understand the mechanism of the canonical Wnt signaling contributing to urethral fibrosis and offered a potential therapeutic target in urethral fibrosis treatment.

## Materials and Methods

### Human urethral fibrosis samples

Human urethral tissues (non-human urethral fibrosis and human urethral fibrosis) were obtained from six male patients with urethral stricture undergoing urethroplasty at The Second Affiliated Hospital of Xi'an Jiaotong University (Xi'an, China). The locations of the strictures were all at the membranous urethra. Clinical samples were collected following surgery and stored at -80°C or fixed in 4% paraformaldehyde solution for further experiments.

### TGFβ1-induced rat urethral fibrosis model

Adult male Sprague-Dawley rats weighing approximately 300 to 350 g (Centre of Laboratory Animals, Xi'an Jiaotong University, Xi'an, China) were maintained under standard conditions with free access to water and laboratory rodent food. After anesthesia with intravenous pentobarbital (30 mg/kg), rats were randomly divided into control group (n = 10), urethral fibrosis group (UF, n = 10) and urethral fibrosis treated with DKK1 group (UF+DKK1, n = 10) for the study. Urethral fibrosis was induced in rats in the UF and UF+DKK1 groups with TGFβ1 local injection and partial incisions as described previously^8^.

Briefly, all the rats were laid in the supine position. After opening the ventral penile skin to visualize the urethral, 10 µg TGFβ1 was injected into the urethral wall at the four positions with a 30-gauge needle and followed with partial incisions of the penile urethra. After 24 h, all the rats were continuously administered a second injection at the same position with either saline (control and UF groups) or 5 mg DKK1 (UF+DKK1 group). All the rats were sacrificed after 4 weeks and the urethral tissues were harvested for further analysis.

### Isolation of human urethral fibroblasts

Human urethral fibroblasts (HUFs) were isolated and cultured as previously described^21^. Briefly, fresh human specimens were obtained from patients with urethral stricture undergoing urethraplasty. The minced urethral tissue was pretreated with 0.2% collagenase (Sigma-Aldrich, St. Louis, MO, USA) for 30 min at 37°C following with centrifugation. Next, these pretreated samples were seeded in DMEM containing 20% fetal bovine serum (Gibco, Grand Island, NY, USA) and 1% penicillin-streptomycin, and maintained in a humidified atmosphere of 95 % air, 5% CO_2_ at 37 °C. The cells surrounding the explants were identified at a mean interval of 10 days after initial plating. HUFs were passaged using 0.25% trypsin when they reached 70-90% confluence. In this study, we established three primary cell lines of human urethral fibroblasts (HUFs) for further experiments.

### Histopathology

The harvested human or rat urethral tissues were fixed in 4% neutral phosphate-buffered paraformaldehyde overnight and processed using routine methods before sectioning into slices. The sections were stained with Hematoxylin-eosin (HE), Masson and Sirius stain, or were subjected to immunohistochemistry analysis. Representative images were captured using a Zeiss Observer I microscope (Zeiss) at magnifications of 20×, 50×, and 100× and acquired using the CaseViewer software.

### Immunohistochemistry staining

Human or rat urethral tissues sections were deparaffinized and incubated in peroxidase solution (3% H_2_O_2_) followed by blocking at room temperature for 30 min with 3 % BSA. Next, slides were incubated with anti-β-catenin antibody (1:200, Abcam), anti-DKK1 antibody (1:500, Abcam) or anti-α-SMA antibody (1:500, Abcam) at 4°C overnight. Slides were then incubated with a second antibody conjugated to horseradish peroxidase (HRP) for 1 h and followed with diaminobenzidine (DAB) staining and neutral resin sealing. The representative images were captured using a Zeiss Observer I microscope (Zeiss) at magnifications of 20×, 50×, and 100× and collected using the CaseViewer software.

### Immunofluorescence staining

HUFs incubated in 24-well plates were fixed in 4% paraformaldehyde for 30 min and permeabilized with 0.5% Triton for 5 min. After blocking in 5% BSA for 1 h, HUFs were incubated with primary antibodies against vimentin (1:200, Abcam), α-SMA (1:200, Abcam) and β-catenin (1:200, Abcam) overnight at 4 °C and subsequently incubated with Alexa Fluor-conjugated secondary antibody for 1 h. HUFs were then incubated with DAPI for 5 min at room temperature and were subsequently detected under Zeiss Observer I fluorescence microscope (Zeiss, Germany).

### Western blot analysis

RIPA buffer was used to extract total protein from HUFs, human and rat urethral tissues. BCA protein assay (Thermo Fisher Scientific) was performed to detect the protein concentration. Equal amounts of protein (20 µg) were separated by 10% SDS-PAGE gels and transferred to PVDF membrane (Millipore, USA). Next, the membranes were blocked in 5% fat-free milk for 2 h, and incubated with the following antibodies at 4 °C overnight: anti-Wnt3a (1:1000, Abcam), anti-β-catenin (1:1000, Abcam), anti-DKK1(1:1000, Abcam), anti-collagen I (1:1000, Abcam), anti-collagen III (1:1000, Abcam), anti-α-SMA (1:1000, Abcam), anti-p-GSK-3β (1:1000, Abcam), anti-GSK-3β(1:1000, Abcam), anti-p-Smad2(1:1000, Abcam), anti-p-Smad3 (1:1000, Abcam), anti-Smad2/3 (1:1000, Abcam) and anti-GAPDH (1:5000, Abcam, Cambridge, UK). Membranes were cut horizontally based on the differences of molecular weight. Then membranes washed with TBST (Beyotime Biotechnology, Shanghai, China) and incubated with corresponding secondary antibodies (goat anti-rabbit IgG H&L (HRP), 1:10000; rabbit anti mouse IgG H&L (HRP), 1:10000; Abcam, USA) at room temperature for 1 h. Additionally, membranes were stripped with Western Blot Stripping Buffer (Cell Signaling Technology), and re-probed with target proteins. Target blots signals were observed by an enhanced chemiluminescence-detecting kit (Thermo Fisher, MA, USA).

### Quantitative real-time polymerase chain reaction (qRT-PCR) assay

By using the Trizol reagents (Thermo Fisher Scientific, Carlsbad, USA), total RNA from the urethral tissues of human, rats and HUFs cells of diverse groups was extracted to conduct reverse transcription. NanoDrop2000 Spectrophotometers (Thermo Fisher Scientific, MA, USA) was utilized to test RNA quality. Expression of mRNA was quantified using PrimeScript RT reagent kit (TaKaRa, Dalian, China) on a a Bio-Rad iQ5 PCR system (Bio-Rad, Hercules, USA). The sequences of primers were listed in Table 1. 2^-ΔΔCt^ method was used to calculate mRNA expression, and GAPDH was served as the internal control.

### Cell growth assay

Cell Counting Kit-8 (CCK-8; Dojindo, Kumamoto, Japan) assay was performed to determine the viability of HUFs after TGFβ1 or DKK1 treatment. Briefly, HUFs were put in a 96-well plate and cultured with TGFβ1 or DKK1 treatment, and then CCK-8 solution (10 μL) was added to each well and the plates were incubated at 37 °C for 2 h, and the optical density (OD) was read at 450 nm absorbance using the ELX-800 Biotek plate reader (Winooski, USA).

### Hydroxyproline assay

Hydroxyproline from collagen was detected using a hydroxyproline assay kit (Bioss, Beijing, China) according to the manufacturer's instructions. Briefly, pretreated HUFs were boiled with 1mL solution for 1 h and neutralized using NaOH. And the cell suspensions were centrifuged for 20 min at 25℃. After that, the supernatant liquids were collected. Measurement at 560 nm absorbance was obtained using the ELX-800 Biotek plate reader (Winooski, USA), and the following formula was used for calculation: hydrolyzed hydroxyproline concentration = B (amount of hydroxyproline)/V (sample volume) × D (dilution factor).

### Cell Apoptosis assay

Cell apoptosis was analyzed by the Annexin V-PI (Beyotime Institute of Biotechnology) staining kit according to manufacturer's instructions. Briefly, after treatment in diverse groups, HUFs were harvested and washed three times with cold PBS, and then were resuspended in 500 μL binding buffer. Afterward, each cell sample (200 μL cell suspension) was added with 5 μL Annexin V-FITC followed by 10 μL propidium iodide for 10 min incubation in the dark at 25 °C for 15 min. Finally, cell apoptosis was performed on a FACS flow cytometer (Becton Dickinson) and were analyzed with FlowJo software (Tree star, Ashland, Oregon).

### Cell migration assay

Cell migration assays were conducted using 8 μM pore size inserts (BD Biosciences, USA). Briefly, pretreated HUFs were seeded in the upper chambers and the lower chambers were filled with complete medium containing 10% fetal bovine serum. And then cells in the chambers were incubated at 37 °C for 24 h. After that, cells in the inserts were with methanol and stained with 0.1% crystal violet (Sigma-Aldrich). A light microscope (Leica DM IL inverted microscope, 10×) was used to count the number of migratory cell and then analyzed using ImageJ software (National Institutes of Health, Bethesda, USA).

### Statistical analysis

All data in this study are presented as mean ± standard error of the mean (SEM) of three independent experiments. GraphPad Prism 9.0 (GraphPad Software, San Diego, USA) was used for statistical analysis.

Comparisons between two groups were conducted by Student's t-test (two-tailed). Comparisons among multiple groups were assessed by one-way analysis of variance (ANOVA) and Tukey's post hoc test. *P*< 0.05 meant statistical significance.

## Results

### Canonical Wnt signaling is activated in urethral fibrosis

**Figure 1:**
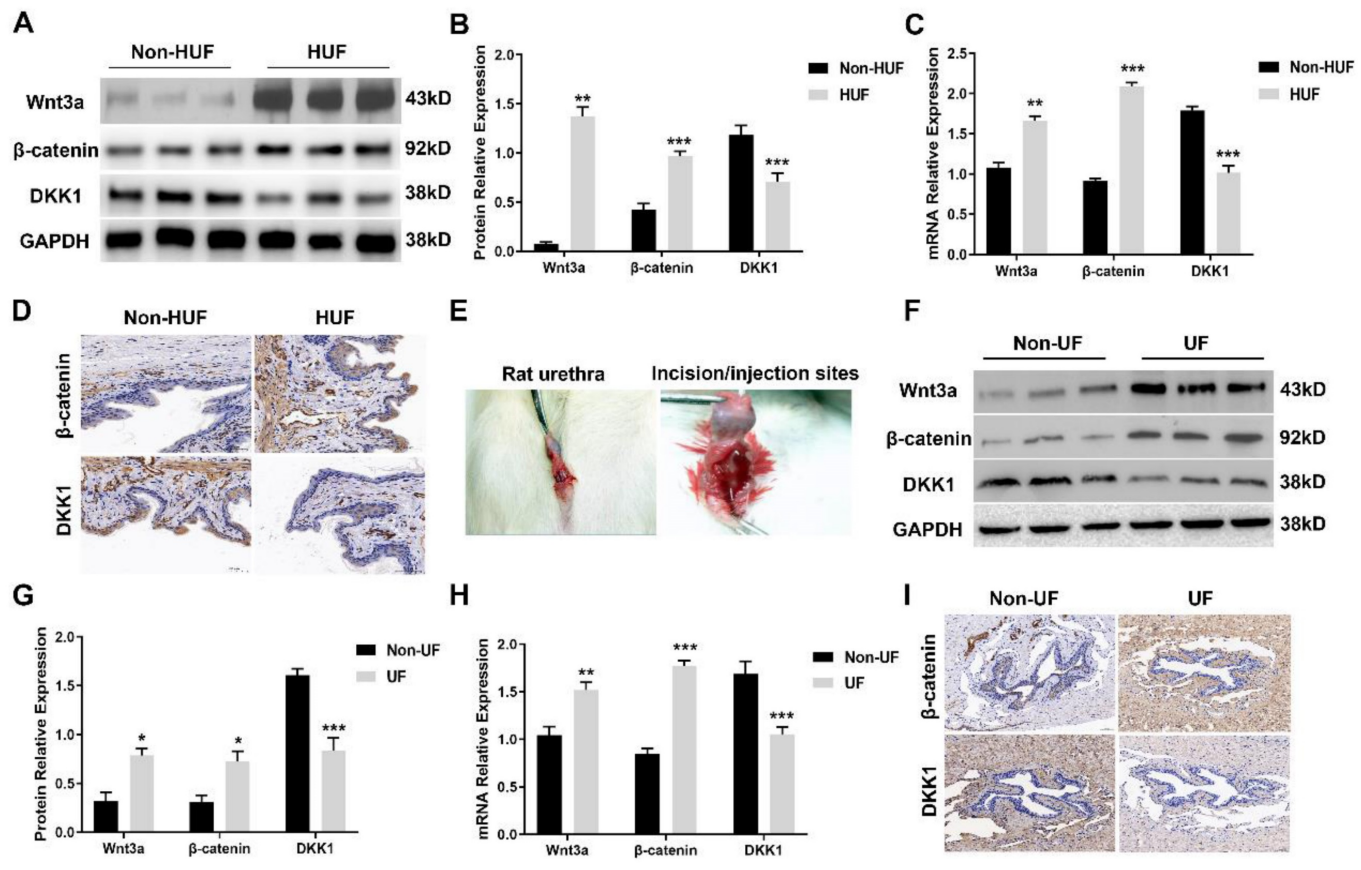
Canonical Wnt signaling is activated in urethral fibrosis disease. (A) Expression of key components of Wnt signaling were examined by western blot in the non-urethral fibrosis (non-HUF) and urethral fibrosis (HUF) groups (n=3for patients with urethral stricture). (B) Quantification of the relative expression of Wnt3a, β-catenin and DKK1 in the non-HUF and HUF groups. (C) Quantitative real-time polymerase chain reaction (qRT-PCR) assays were used to measure the mRNA expression levels of Wnt3a, β-catenin and DKK1 in the non-HUF and HUF groups. (D) The content of β-catenin and DKK1 in the non-HUF and HUF groups were detected through immunohistochemistry at magnifications of 100×. (E) Representative images of urethra fibrosis rat model. (F) Expression of Wnt3a, β-catenin and DKK1 were measured by western blot in the non-urethral fibrosis (non-UF) and urethral fibrosis (UF) groups (n=5 rats for each group). (G) Quantification of the relative expression of Wnt3a, β-catenin and DKK1 in the non-UF and UF groups. (H) Quantitative real-time polymerase chain reaction (qRT-PCR) assays were used to measure the mRNA expression levels of Wnt3a, β-catenin and DKK1 in the non-UF and UF groups. (I) The content of β-catenin and DKK1 in the non-UF and UF groups were detected through immunohistochemistry at magnifications of 100×. Data are expressed as mean ± SEM (n = 3, non-HUF group; n = 3, HUF group; n = 5, non-UF group; n = 5, UF group). * *P* < 0.05, ** *P* < 0.01, *** *P* < 0.001. non-HUF group vs. HUF group; non-UF group vs. UF group.

To investigate the activation of the canonical Wnt cascade, we first assessed Wnt3a, β-catenin and the endogenous inhibitor DKK1 expression level in human urethral fibrosis. Human urethra tissues derived from patients with urethral stricture were divided into two groups: non-human urethral fibrosis (non-HUF) and human urethral fibrosis (HUF). The protein levels of Wnt3a and β-catenin were strongly expressed in the HUF groups compared to the non-HUF groups (Figure 1A, B). In the addition to the upregulation of Wnt3a and β-catenin, the expression of DKK1 was strongly decreased in the HUF groups (Figure 1A, B). Consistently, the mRNA levels of Wnt3a and β-catenin were significantly increased and DKK1were decreased in the HUF groups compared to the non-HUF groups (Figure 1C). Furthermore, human urethra sections of the two groups were stained for β-catenin and DKK1. As shown in Figure 1D, abundance of β-catenin was detected in the HUF groups as compared with the non-HUF groups, whereas the expression of DKK1 was rarely absent in the HUF groups. Having observed the activation of canonical Wnt signaling in human urethral fibrosis, we next used a rat model of urethral stricture with local urethral incision and TGFβ1 injection to investigate whether the canonical Wnt signaling is activated (Figure 1E). Urethra tissues of rats were derived from two groups: non-urethral fibrosis (non-UF) and urethral fibrosis (UF). Consistent with the finding in human urethral fibrosis, the protein and mRNA levels of Wnt3a and β-catenin were significantly increased in the UF groups, whereas the levels of DKK1 was notably decreased in the UF groups compared to the non-UF groups (Figure 1F, H). In parallel to the results of Western blot and real-time PCR, a increased staining of β-catenin and decreased staining of DKK1 was observed in the UF groups compared to the non-UF groups (Figure 1I). Together, these results indicated that canonical Wnt signaling is activated in urethral fibrosis and overexpression of the endogenous inhibitor DKK1 could be evaluated as a potential therapeutic target for urethral fibrosis.

### The endogenous inhibitor DKK1 ameliorates TGFβ1-induced urethral fibrosis in rats

**Figure 2:**
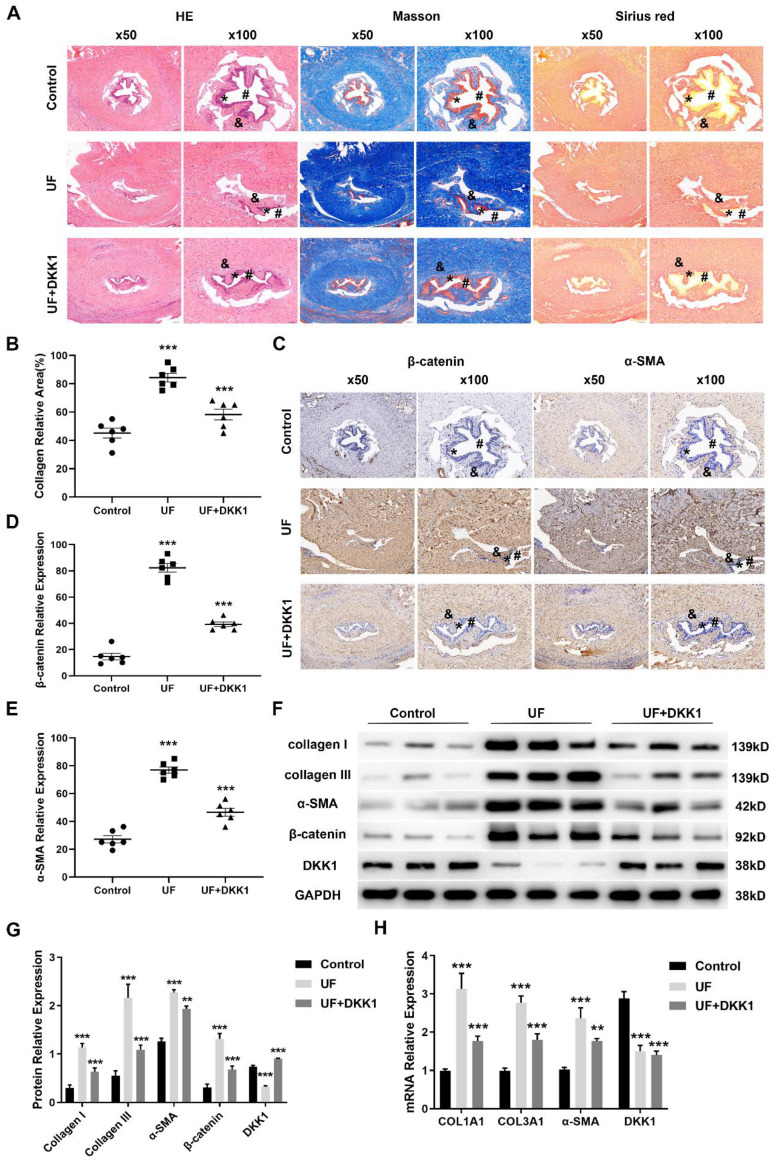
The endogenous inhibitor DKK1 ameliorates TGFβ1-induced urethral fibrosis in rats. (A) Representative histologic images of Hematoxylin-eosin (HE), Masson and Sirius stained midshaft sections of rat penises at magnifications of 50× and 100× in the control groups, UF groups and UF+DKK1 groups. (urethral lumen (#), urethral epithelium (*), urethral fibrosis (&)). (B) Quantitative analysis of the relative collagen area in the control groups, UF groups and UF+DKK1 groups. (C) The content of β-catenin and α-SMA in the control groups, UF groups and UF+DKK1 groups were detected by immunohistochemistry. (D, E) Quantitative analysis of the relative expression of β-catenin and α-SMA in the control groups, UF groups and UF+DKK1 groups. (F) Expression of key components of Wnt signaling and fibrotic markers were examined by western blot in the control groups, UF groups and UF+DKK1 groups (n=10 rats for each group). (G) Quantification of the relative expression of Wnt3a, β-catenin and DKK1 in the control groups, UF groups and UF+DKK1 groups. (H) qRT-PCR assays were used to measure the mRNA expression levels of COL1A1, COL3A1, α-SMA and DKK1 in the control groups, UF groups and UF+DKK1 groups. Data are expressed as mean ± SEM (n = 10, control group; n = 10, UF group; n = 10, UF+DKK1 group). ** *P* < 0.01, *** *P* < 0.001. control group vs. UF group; UF group vs. UF+DKK1 group.

We next investigated whether inhibition of canonical Wnt signaling blocks urethral fibrosis. Having demonstrated that the endogenous inhibitor DKK1 is significantly downregulated in urethral fibrosis, overexpression of DKK1 was chosen to target canonical Wnt signaling. We therefore performed rat model of urethral stricture and separated them into three groups: control groups, UF groups and UF treated with DKK1 groups (UF+DKK1). Histology of rat urethral tissue sections of the three groups were shown as in Figure 2A (urethral lumen (#), urethral epithelium (*), urethral fibrosis (&)). According to the results of Hematoxylin-eosin (HE), Masson and Sirius staining, normal urethral lumen was lined by a layer of pseudo-stratified columnar epithelium and also was surrounded by normal distribution of collagen bundles without fibrosis in the control groups. In contrast, there was a narrow urethral lumen and moderate fibrosis with deposition of amorphic extracellular matrix (densely packed collagen bundles) beneath the urethral epithelium in the UF groups. Importantly, with treatment of DKK1, only mild fibrosis (less disorganization of extracellular matrix) and fewer collagen bundle depositions were observed in the UF+DKK1 groups. The quantitative analysis showed the significant increase of collagen area in the UF groups and decrease of collagen in the UF+DKK1 groups based on the results of Masson staining (Figure 2B). Moreover, the adjacent sections from the same rat in the three groups were stained for β-catenin and α-SMA. Abundance of β-catenin and α-SMA were detected in the UF groups as compared with the control groups, whereas their levels were significantly decreased with DKK1 treatment as compared with the UF groups (Figure 2C, E). We further assessed a number of markers associated with fibrosis and key factors of canonical Wnt signaling. As presented in Figure 2F, G, the UF groups significantly upregulated protein expression of collagen I, collagen III, α-SMA and β-catenin and downregulated protein expression of DKK1 as compared with the control group, and the expression was effectively inhibited by DKK1 treatment as compared with the UF groups, while the DKK1 expression was rescued by DKK1 treatment in the UF+DKK1 groups (Figure 2F, G). Comparable results were obtained when COL1A1, COL3A1 and α-SMA were analyzed at the transcription level (Figure 2H). Interestingly, DKK1 treatment significantly reduced DKK1 mRNA levels in UF groups but showed no effects in UF+DKK1 groups as DKK1 was exogenous.

### Canonical Wnt signaling is activated during myofibroblast differentiation of human urethral fibroblasts

**Figure 3:**
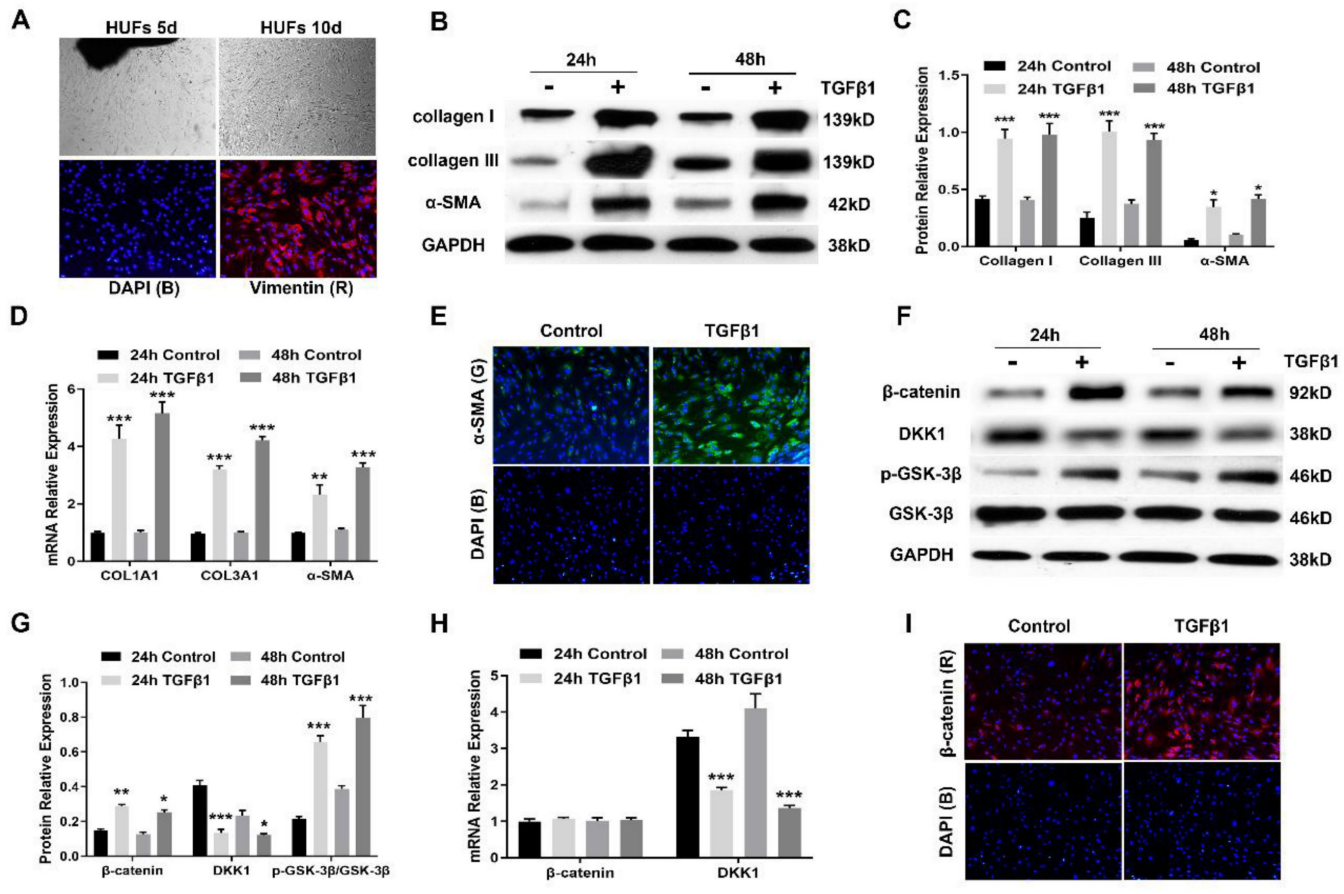
Canonical Wnt signaling is activated during myofibroblast differentiation of human urethral fibroblasts. (A) Representative images of established primary human urethral fibroblasts (HUFs) from fresh surgical urethral specimens isolated from patients with urethral fibrosis under a light microscope (20×) and vimentin positive staining (Red), nuclear DAPI staining (Blue)under a fluorescent microscope (40×). (B) Western blot analysis of collagen I, collagen III and α-SMA protein expression in HUFs with or without TGFβ1 (10 ng/mL) stimulation for 24h or 48h. (C) Quantification of the relative expression of collagen I, collagen III and α-SMA in HUFs with or without TGFβ1 (10 ng/mL) stimulation for 24h or 48h. (D) qRT-PCR analysis of COL1A1, COL3A1 and α-SMA mRNA expression in HUFs with or without TGFβ1 (10 ng/mL) stimulation for 24h or 48h. (E) The content of α-SMA staining (Green) in HUFs with TGFβ1 (10 ng/mL) treatment were detected by immunofluorescence staining at magnifications of 20×. (F) The central signaling components of the canonical Wnt pathway were measured by western blot in HUFs with or without TGFβ1 (10 ng/mL) stimulation for 24h or 48h. (G) Quantification of the relative expression of β-catenin, DKK1 and p-GSK-3β in HUFs with or without TGFβ1 (10 ng/mL) stimulation for 24h or 48h. (H) qRT-PCR analysis of β-catenin and DKK1 mRNA expression in HUFs with or without TGFβ1 (10 ng/mL) stimulation for 24h or 48h. (I) The content of β-catenin staining (Red) in HUFs with TGFβ1 (10 ng/mL) treatment were detected by immunofluorescence staining at magnifications of 20×. Data are expressed as mean ± SEM (n = 3). * *P* < 0.05, ** *P* < 0.01, *** *P* < 0.001. 24h control group vs. 24h TGFβ1 group; 48h control group vs. 48h TGFβ1 group.

Having demonstrated that aberrant activation of canonical Wnt signaling is found in urethral fibrosis, we next established primary human urethral fibroblasts (HUFs) to investigate whether canonical Wnt signaling is activated during myofibroblast differentiation. For HUFs, cell suspensions from human urethral tissues plated in culture medium gave rise to multiple plastic-adherent fibroblast cells after approximately 10 days, which were expanded by subsequent trypsinization and several rounds of passaging (Figure 3A). These urethra-derived adherent cells displayed the morphology of a typical spindle, spiral and radial arrangement, which were identified by positive vimentin staining (Figure 3A). Following treatment with TGFβ1 at 10 ng/mL for 24h and 48h respectively, HUFs displayed increased expression of collagen I, collagen III and α-SMA, which were the major marker of myofibroblasts (Figure 3B, C). Similarly, the mRNA expression of several major profibrotic genes was examined by real-time PCR. mRNA for COL1A1, COL3A1 and α-SMA were upregulated by TGFβ1 treatment (Figure 3D). Furthermore, accumulation of α-SMA was tested by immunofluorescence staining (Figure 3E). The expression of canonical Wnt signaling components during the myofibroblast differentiation of HUFs was also assessed. Western blot revealed upregulation of β-catenin and p-GSK-3β in HUFs after incubation with TGFβ1, while stimulation with TGFβ1 reduced the protein levels of DKK1(Figure 3F, G). Interestingly, expression of β-catenin was not affected by TGFβ1 treatment at the transcription level, while the mRNA levels of DKK1 was decreased after incubation with TGFβ1 (Figure 3H). Due to the decreased expression of DKK1, it indicated that TGFβ signaling might regulate canonical Wnt signaling via DKK1. Moreover, accumulation of β-catenin was analyzed by immunofluorescence staining (Figure 3I). Taken together, TGFβ1 treatment induced myofibroblast differentiation along with the activation of canonical Wnt signaling in HUFs.

### Inhibition of canonical Wnt signaling with DKK1 prevents the stimulatory effects of TGFβ1 on HUFs

**Figure 4:**
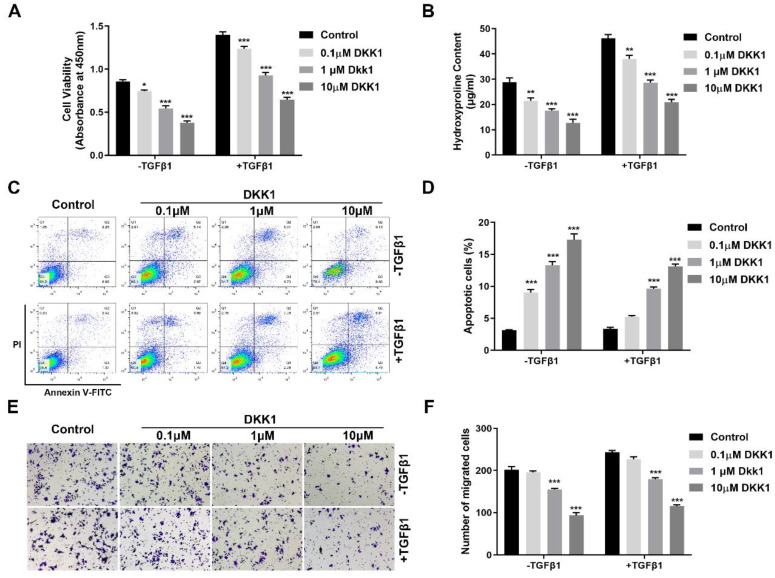
Inhibition of canonical Wnt signaling with DKK1 prevents the stimulatory effects of TGFβ1 on HUFs. (A) Cell Counting Kit (CCK-8) assay was performed to determine the viability of HUFs after treatment with DKK1 at various concentrations in the presence or absence of TGF-β1 (10 ng/ml) for 24h. (B) Hydroxyproline content assay was used to detect the collagen content of HUFs after treatment with DKK1 at various concentrations in the presence or absence of TGF-β1 (10 ng/ml) for 24h. (C, D) Cell apoptosis of HUFs after treatment with DKK1 at various concentrations in the presence or absence of TGF-β1 (10 ng/ml) for 24h was evaluated by flow cytometry measuring annexin V and propidium iodide (PI) expression.( E, F) Cell migration assay of HUFs after treatment with DKK1 at various concentrations in the presence or absence of TGF-β1 (10 ng/ml) for 24h. Data are expressed as mean ± SEM (n = 3). * *P* < 0.05, ** *P* < 0.01, *** *P* < 0.001. control group vs. DKK1 group; control group (TGFβ1) vs. TGFβ1+DKK1 group.

To determine whether overexpression of DKK1 blocks the stimulatory effects of TGFβ1 on HUFs, we firstly evaluated the cell proliferation of TGFβ1 stimulation in the presence of DKK1.DKK1 significantly suppressed cell proliferation of HUFs in the absence or presence of TGFβ1 in a dose-dependent manner (Figure 4A). Then we assessed the effect of DKK1 on collagen production by testing hydroxyproline content, TGFβ1 increased hydroxyproline content of HUFs while DKK1 significantly reduced it with or without TGFβ1 treatment in a dose-dependent manner (Figure 4B). Furthermore, Annexin V-FITC/PC assay was used for the effects of DKK1 on the apoptosis of HUFs. The percentage of apoptotic cells was significantly increased by DKK1 while attenuated by TGFβ1 in a dose-dependent manner (Figure 4C, D), indicating the pro-survival effects of TGFβ1 on HUFs. Moreover, we also investigated the effect of DKK1 on the migration of HUFs. Cell migration was clearly promoted by TGFβ1 but remarkably inhibited by DKK1 in a dose-dependent manner (Figure 4E, F). These data indicated that inhibition of canonical Wnt signaling with DKK1 suppresses cell proliferation, collagen production and cell migration and promotes cell apoptosis of TGFβ1-induced HUFs.

### Inhibition of canonical Wnt signaling with DKK1 inhibits TGFβ1-induced myofibroblasts differentiation of HUFs by a Smad-independent mechanism

**Figure 5:**
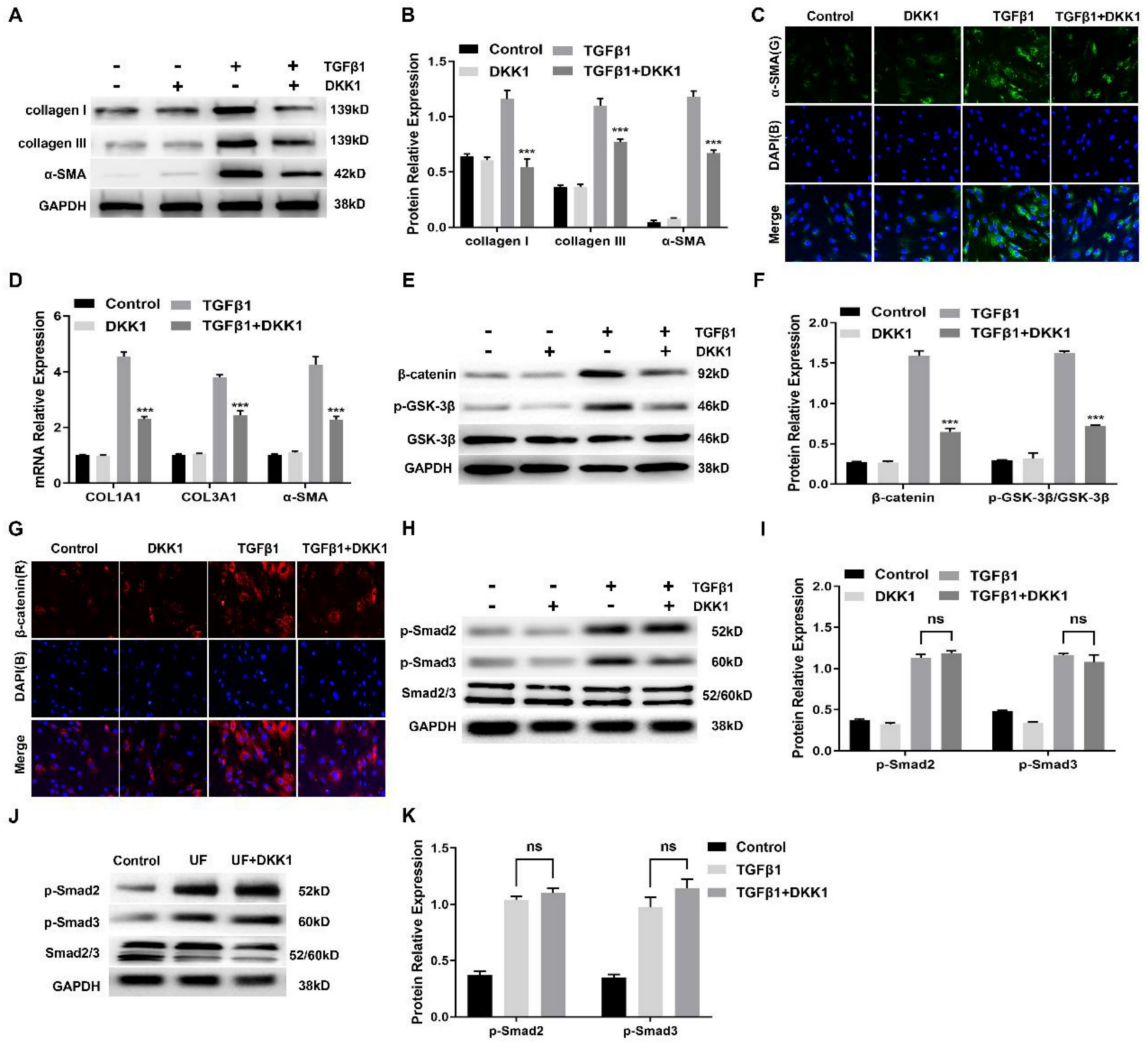
Inhibition of canonical Wnt signaling with DKK1 inhibits TGFβ1-induced myofibroblasts differentiation of HUFs by a Smad-independent mechanism. (A) Western blot analysis of collagen I, collagen III and α-SMA protein expression in HUFs with DKK1 treatment in the presence or absence of TGF-β1 (10 ng/ml) for 24h. (B) Quantification of the relative expression of collagen I, collagen III and α-SMA in HUFs with DKK1 treatment in the presence or absence of TGF-β1 (10 ng/ml) for 24h. (C) The content of α-SMA in HUFs with DKK1 treatment in the presence or absence of TGF-β1 (10 ng/ml) for 24h were detected by immunohistochemistry at magnifications of 20×. (D) qRT-PCR analysis of COL1A1, COL3A1 and α-SMA mRNA expression in HUFs with DKK1 treatment in the presence or absence of TGF-β1 (10 ng/ml) for 24h. (E) Western blot analysis of β-catenin and p-GSK-3β in HUFs with DKK1 treatment in the presence or absence of TGF-β1 (10 ng/ml) for 24h. (F) Quantification of the relative expression of β-catenin and p-GSK-3β in HUFs with DKK1 treatment in the presence or absence of TGF-β1 (10 ng/ml) for 24h. (G) The content of β-catenin in HUFs with DKK1 treatment in the presence or absence of TGF-β1 (10 ng/ml) for 24h were detected by immunohistochemistry at magnifications of 20×. (H) Western blot analysis of p-Smad2, p-Smad3 and Smad2/3 in HUFs with DKK1 treatment in the presence or absence of TGF-β1 (10 ng/ml) for 24h. (I) Quantification of the relative expression of p-Smad2/Smad2/3 and p-Smad3/Smad2/3 in HUFs with DKK1 treatment in the presence or absence of TGF-β1 (10 ng/ml) for 24h. (J) Western blot analysis of p-Smad2, p-Smad3 and Smad2/3 in the control groups, UF groups and UF+DKK1 groups. (K) Quantification of the relative expression of p-Smad2/Smad2/3 and p-Smad3/Smad2/3 in the control groups, UF groups and UF+DKK1 groups. Data are expressed as mean ± SEM (n = 3). *** *P* < 0.001. ns: no significant difference. D1 group vs. TGFβ1+DKK1 group; UF group vs. UF+DKK1 group.

Having observed a critical role for canonical Wnt signaling activation during myofibroblast differentiation of HUFs, we further assessed whether targeted inhibition of this signaling could reduce profibrotic events in TGFβ1-induced HUFs. We analyzed a number of markers related to fibrosis and extracellular matrix. As shown in Figure 5A, B, TGFβ1 significantly promoted protein levels of collagen I, collagen III and α-SMA in HUFs, while expression was effectively suppressed by DKK1. Immunofluorescence staining also confirmed that the content of α-SMA in TGFβ1-induced HUFs was inhibited by DKK1 (Figure 5C). Consistently, similar results of COL1A1, COL3A1 and α-SMA at the transcription level were significantly down-regulated by DKK1 in TGFβ1-induced HUFs (Figure 5D). As key components of canonical Wnt signaling, protein levels of β-catenin and p-GSK-3β were significantly upregulated by TGFβ1 while inhibited by DKK1 (Figure 5E, F). Meanwhile, abundant β-catenin was detected in the TGFβ1-induced HUFs while it was decreased by DKK1 (Figure 5G). Importantly, Smad2 and Smad3 phosphorylation were not affected by DKK1 in HUFs while were significantly upregulated in the TGFβ1-induced HUFs (Figure 5H, I), suggesting DKK1 disrupted myofibroblasts differentiation of TGFβ1-induced HUFs by independence of the canonical Smad pathway. Furthermore, DKK1 also exhibited no effect on Smad2 and Smad3 phosphorylation in the treated with DKK1 groups (Figure 5J, K), indicating that DKK1 also exerts its anti-fibrosis by a mechanism independent of intervention of Smad activation *in vivo*.

## Discussion

Urethral stricture, a common disorder of the lower urinary tract in men with unknown etiology, is characterized by progressive narrowing of urethral lumen. The key histological feature of urethral fibrosis is the accumulation of fibroblasts and excessive collagen deposition^22^. In addition, activation of fibroblasts and their differentiation into myofibroblasts are the central event of urethral fibrosis formation^23^. However, owing to the complex network of interactions and many compensatory pathways involved in urethral fibrosis, the molecular mechanisms of urethral fibrosis leading to abnormal activation of fibroblasts and persistently increased release of extracellular matrix components are incompletely understood.

To date, very few effective anti-fibrotic agents are currently available for patients with urethral stricture. In this study, we mainly focused on the potential role of canonical Wnt signaling in urethral fibrosis and aimed to explore novel potential targets for preventing urethral fibrosis. It is well documented that pathologically activated canonical Wnt signaling occurs in various fibrotic diseases^13^. However, it is still unknown whether canonical Wnt signaling is associated with the process of urethral fibrosis. Indeed, some studies have reported that Wnt/β-catenin signaling inhibitors could inhibit urethral fibrosis, but still lacking evidence that canonical Wnt signaling contributes to urethral fibrosis^24^, ^25^. We observed an overexpression of the Wnt proteins Wnt3a, β-catenin and also a significantly decreased expression of the the endogenous inhibitor DKK1 in human urethra tissues derived from patients with urethral stricture. By establishing a rat model of urethral fibrosis, we demonstrate that the canonical Wnt signaling was activated.

As a crucial role of the canonical Wnt signaling and its potential profibrotic effects, we reasoned that inhibiting this signaling might result in preventing urethral fibrosis. Accumulating evidence indicates that increased activation of canonical Wnt signaling might have an important role in fibrogenesis. To avoid uncontrolled activation, canonical Wnt signaling is tightly controlled by an array of negative regulators. DKK1 is one of the negative regulators. DKK1 expression level was reduced in urethral fibrosis based on our study results consistent with other fibrotic diseases. Considering the decreased levels of the endogenous inhibitor DKK1, we have chosen overexpression of DKK1 to inhibit the canonical Wnt pathway. DKK1 inhibits the canonical Wnt signaling by binding to the LRP5/6 co-receptor and blocking Wnt binding, which results in β-catenin degradation. The reason why we chose DKK1 as a potential target to prevent urethral fibrosis is that it's already reported that decreased levels of DKK1 contributed to the activation of the canonical Wnt pathway in human fibrosis and is currently investigated as a potential therapeutic target in other diseases^26^, ^27^. Numerous studies have shown that DKK1 plays a critical role in fibrosis diseases and could serve as a potential therapeutic target in fibrotic diseases, including pulmonary fibrosis, renal fibrosis, liver fibrosis and systemic sclerosis^28^. In addition, DKK1 has a broader inhibition of the canonical Wnt pathway than targeting Wnt proteins^20^. In our study, upon the treatment of DKK1, TGFβ1-induced urethral fibrosis in rats were ameliorated with decreasing of excessive collagen deposition.

At the cellular level, it is currently demonstrated that fibroblasts play an important role in the process of fibrosis^29^. By isolating human urethral fibroblasts following incubation with TGFβ1, we indicate that the canonical Wnt signaling was activated and was able to transform fibroblasts into myofibroblasts evidenced by the expression of specific markers of myofibroblasts. Of particular interest, our results reveal the crosstalk between TGFβ signaling and the canonical Wnt pathway by the upregulation of β-catenin and p-GSK-3β and downregulation of DKK1 in TGFβ1-induced human urethral fibroblasts. In human fibroblasts, TGFβ stimulates canonical Wnt signaling in a p38-dependent manner by inducing nuclear accumulation of β-catenin and reducing the expression of DKK1^13^.Thus, TGFβ1 seems to be the major factor for the activation of the canonical Wnt pathway in fibrotic diseases. However, direct targeting of TGFβ is unlikely to be a therapeutic target as the involvement of TGFβ1 in other systems, like the immune system^30^. In addition, inhibition of downstream pathways of TGFβ can't completely disrupt the stimulatory effects of TGF-β on fibroblasts, indicating that other additional pathways are important for contributing to the profibrotic effects of TGFβ^13^. In this study, we observed the effects of DKK1 on cellular functions, including cell proliferation, collagen production, cell migration and cell apoptosis. Interestingly, DKK1 inhibited cell proliferation, collagen production, cell migration and promoted cell apoptosis in TGFβ1-induced human urethral fibroblasts. These results have an important effect on fibroblast functions which can explain why DKK1 can inhibit urethral fibrosis at the cellular level. Further, we explored the potential molecular mechanisms that DKK1 could inhibit the urethral fibrosis by blocking the canonical Wnt signaling. Excessive extracellular matrix deposition is considered as a critical process in urethral fibrosis^31^. Emerging evidence has shown that collagen I and collagen III are major components of extracellular matrix in urethral fibrosis^32^. Therefore, our results revealed that inhibition of canonical Wnt signaling with DKK1 inhibits expression of collagen I, collagen III and α-SMA in TGFβ1-induced human urethral fibroblasts.

Of note, suppression of DKK1 in TGFβ1-induced human urethral fibroblasts was independent of Smad2 and Smad3, indicated that these actions are performed by a mechanism independent of any disruption of Smad activation. Consistently, similar findings were found* in vivo.* TGFβ is a key regulator of fibroblast activation in fibrotic diseases. Smad proteins are considered as major signaling intermediaries for the activation of TGFβ in fibroblasts. However, the effects of TGFβ are mediated by a complex network of intracellular signaling. Smad-indepent signals have also been implicated in the profibrotic effects of TGFβ. As we know, DKK1 inhibits the canonical Wnt signaling by binding to the LRP5/6 co-receptor and blocking Wnt binding, which results in β-catenin degradation. Our results have confirmed that DKK1 could directly reduce β-catenin and p-GSK-3β expression level to block Wnt signaling in Figure 5E. This could explain how DKK1 exert its antifibrotic functions. Previous study has been reported that the TGFβ-mediated suppression of DKK1 was independent of Smad proteins but was mediated by MAPK p38^13^. Besides, TGFβ could stimulate Wnt signaling by inhibition of GSK-3β aside decreasing DKK1in normal and tumoral fibroblasts^33^. Therefore, we performed experiments to confirm that whether the antifibrotic effect of DKK1 involve interference with Smad signaling. And our results indicated that DKK1 exerts its anti-fibrosis by a mechanism independent of intervention of Smad proteins, which was consistent with these studies. And these results also suggested that Wnt signaling is important for transmission of the profibrotic effects of TGFβ.

There was also a limitation in this study, that was we did not have a directly method to assess the degree of urethral lumen stenosis in a rat model of urethral fibrosis. Therefore, future studies should include retrograde urethrography or micro-ultrasound to have a better understanding of urethral fibrosis with DKK1 treatment.

## Conclusion

In conclusion, our studies provided evidence to better understand the potential roles of the canonical Wnt signaling in urethral fibrosis as an important mechanism mediating fibroblasts activation and fibrogenesis. In addition, DKK1 contributed to inhibit urethral fibroblatsts differentiation into myofibroblasts by downregulating β-catenin and downstream target genes which might guide the way to explore the potential targets to prevent urethral fibrosis.

## Supplementary Material

Supplementary figures.

## Figures and Tables

**Table 1:** Primer Sequence

	Genes	Forward primers	Reverse primers
Human	Wnt3a	AGCTACCCGATCTGGTGGTC	CAAACTCGATGTCCTCGCTAC
	β-catenin	ATGGCCATGGAACCAGACAG	CAGGGAACATAGCAGCTCGT
	DKK1	GAGCTACCCGGGTCTTTGTC	GGGTACGGCTGGTAGTTGTC
	COL1A1	GCCAAGACGAAGACATCCCA	GGCAGTTCTTGGTCTCGTCA
	COL3A1	GGCTACTTCTCGCTCTGCTT	GTGGGCAAACTGCACAACAT
	α-SMA	ACTGCCTTGGTGTGTGACAA	TCCCAGTTGGTGATGATGCC
	GAPDH	GGTCACCAGGGCTGCTTTTA	TTCCCGTTCTCAGCCTTGAC
Rat	Wnt3a	ACAGGGCACTAACAAGTCGG	ACAGAGAATGGGCTGAGTGC
	β-catenin	ATGGAGCCAGACAGAAAGGC	CTTGCCACTCAGGGAAGGAG
	DKK1	GGTCGTGCTTTCAACGATGG	AGGGTAGGGCTGGTAGTTGT
	COL1A1	AGCTGCATACACAATGGCCT	CTTGGGTCCCTCGACTCCTA
	COL3A1	TGCAATGTGGGACCTGGTTT	GGGCAGTCTAGTGGCTCATC
	α-SMA	GGCTTTGCTGGTGATGATGC	TCCCAGTTGGTGATGATGCC
	GAPDH	ACTACAAACCCAGGAGGGGT	GATGGTGATGGGTTTCCCGT

## Abbreviations

TGFβ: transforming growth factor beta
IHC: immunohistochemistry
IF: immunofluorescence
UF: urethral fibrosis
non-UF: non-urethral fibrosis
non-HUF: non-human urethral fibrosis
HUF: human urethral fibrosis
DKK1: Dickkopf1
HUFs: human urethral fibroblast
α-SMA: alpha smooth muscle actin
CCK-8: Cell Counting Kit-8
COL1A1: collagen type I alpha 1 chain
RT-qPCR: quantitative reverse transcription polymerase chain reaction
Smad2: mothers against decapentaplegic homolog 2
Smad3: mothers against decapentaplegic homolog 3
