# Supplementary material for: DKK1 Ameliorates Myofibroblast Differentiation in Urethral Fibrosis in Vivo and in Vitro by Regulating the Canonical Wnt Pathway
Source: Int J Med Sci. 2023 Sep 25;20(12):1631–43. doi: 10.7150/ijms.79827 (PMC10583189; doi:10.7150/ijms.79827)

Figure S1 (A) Expression of key components of Wnt signaling were examined by western blot in the non-urethral fibrosis (non-HUF) and urethral fibrosis (HUF) groups (n=3 for patients with urethral stricture). The other two results of repeated experiments were shown here.

Figure S2 The other two results of repeated experiments of representative histologic images of Hematoxylin-eosin (HE), Masson stained midshaft sections of rat penises at magnifications of 50×, and 100× in the control groups, UF groups and UF+DKK1 groups. (urethral lumen (#), urethral epithelium (*), urethral fibrosis (&)).

Figure S1

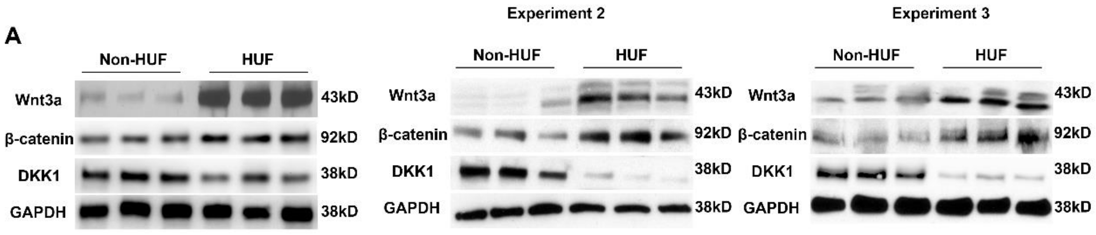

FigureS2

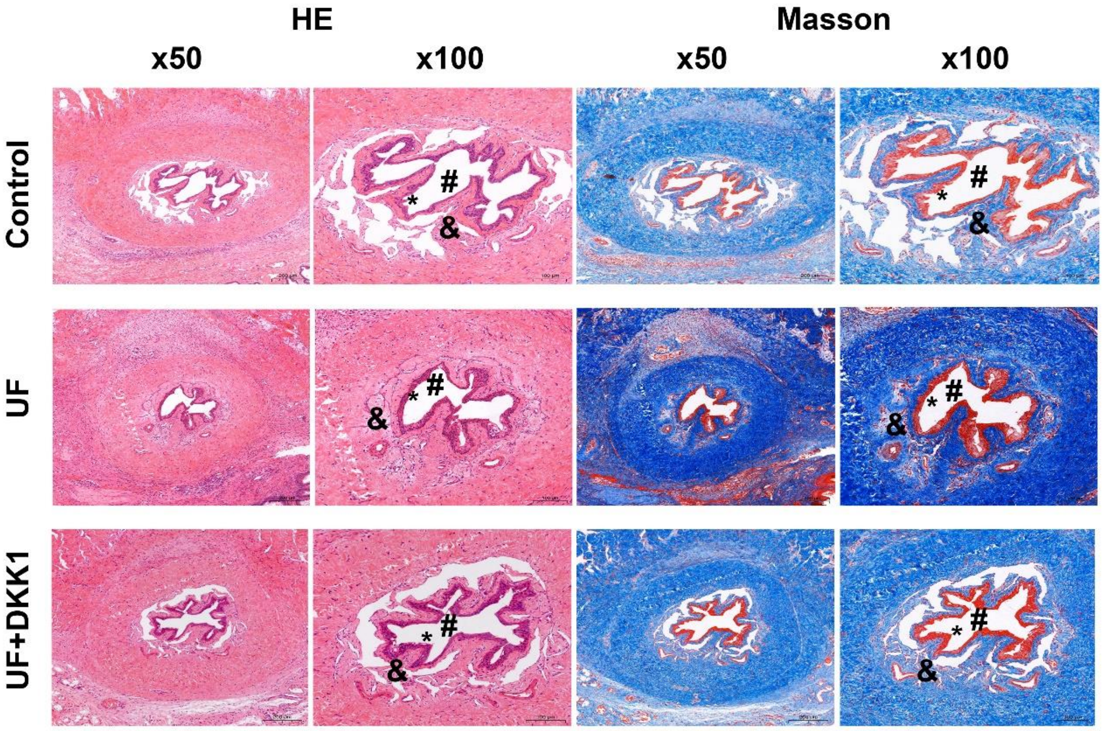

Supplement: Supplementary file 1 — Supplementary figures. [file ijmsv20p1631s1.pdf]
